# *Methanosarcina acetivorans* C2A Topoisomerase IIIα, an Archaeal Enzyme with Promiscuity in Divalent Cation Dependence

**DOI:** 10.1371/journal.pone.0026903

**Published:** 2011-10-26

**Authors:** Raymond Morales, Palita Sriratana, Jing Zhang, Isaac K. O. Cann

**Affiliations:** 1 Department of Biochemistry, University of Illinois, Urbana, Illinois, United States of America; 2 Department of Microbiology, University of Illinois, Urbana, Illinois, United States of America; 3 Department of Animal Sciences, University of Illinois, Urbana, Illinois, United States of America; 4 Institute for Genomic Biology, University of Illinois, Urbana, Illinois, United States of America; Max-Planck-Institute for Terrestrial Microbiology, Germany

## Abstract

Topoisomerases play a fundamental role in genome stability, DNA replication and repair. As a result, topoisomerases have served as therapeutic targets of interest in Eukarya and Bacteria, two of the three domains of life. Since members of Archaea, the third domain of life, have not been implicated in any diseased state to-date, there is a paucity of data on archaeal topoisomerases. Here we report *Methanosarcina acetivorans* TopoIIIα (MacTopoIIIα) as the first biochemically characterized mesophilic archaeal topoisomerase. Maximal activity for MacTopoIIIα was elicited at 30–35°C and 100 mM NaCl. As little as 10 fmol of the enzyme initiated DNA relaxation, and NaCl concentrations above 250 mM inhibited this activity. The present study also provides the first evidence that a type IA Topoisomerase has activity in the presence of all divalent cations tested (Mg^2+^, Ca^2+^, Sr^2+^, Ba^2+^, Mn^2+^, Fe^2+^, Co^2+^, Ni^2+^, Cu^2+^, Zn^2+^ and Cd^2+^). Activity profiles were, however, specific to each metal. Known type I (ssDNA and camptothecin) and type II (etoposide, novobiocin and nalidixic acid) inhibitors with different mechanisms of action were used to demonstrate that MacTopoIIIα is a type IA topoisomerase. Alignment of MacTopoIIIα with characterized topoisomerases identified Y317 as the putative catalytic residue, and a Y317F mutation ablated DNA relaxation activity, demonstrating that Y317 is essential for catalysis. As the role of Domain V (C-terminal domain) is unclear, MacTopoIIIα was aligned with the canonical *E. coli* TopoI 67 kDa fragment in order to construct an N-terminal (1–586) and a C-terminal (587–752) fragment for analysis. Activity could neither be elicited from the fragments individually nor reconstituted from a mixture of the fragments, suggesting that native folding is impaired when the two fragments are expressed separately. Evidence that each of the split domains plays a role in Zn^2+^ binding of the enzyme is also provided.

## Introduction

Topoisomerases manage DNA supercoiling and are classified according to the mechanism of action employed [1]–[4]. In general, topoisomerases form a phosphotyrosine intermediate while cleaving one strand (type I or odd-numbered) or two strands (type II or even-numbered) of DNA. Before the initial transesterification is reversed and a ligated DNA backbone regenerated, type I and II enzymes change the DNA linking number (Lk) by 1 and 2, respectively. Type I topoisomerases are ascribed further distinction based on the polarity of the covalent intermediate formed. Type IA (like Type II) forms a 5′-phosphotyrosine adduct while type IB forms 3′-phosphotyrosine covalent adduct. Thus, the structure and mechanism of each class are distinct.

The characterization of the first topoisomerase *E. coli* TopoI (EcoTopoI), originally titled ω protein, was an earnest exploration into a field that has grown to be of burgeoning biological importance [5]. Since then, topoisomerases have been demonstrated to be vital components of the cellular machinery in a wide array of processes that are essential for life, such as DNA replication, DNA repair and chromosome segregation [6]–[8]. While these molecular machines have been identified in every genome sequenced to date, irrespective of domain, eukaryotic and bacterial topoisomerases have received greater scrutiny given their role in diseased states such as Cancer and infections [9], [10].

Interestingly, studies from bacteria and both lower and higher eukaryotes have demonstrated that ablation of TopoIII (a subfamily of type IA) activity *in vivo* without some compensatory mechanism leads to genomic instability and/or abnormal growth [11]–[16]. In spite of this direct evidence that TopoIII is vital for normal development, the majority of archaeal studies have focused on the non-essential Reverse Gyrase (a subfamily of type IA) given its unique phylogenetic distribution in thermophiles and hyperthermophiles [17]–[20]. Furthermore, the only data published on archaeal TopoIII are from hyperthermophilic organisms of the archaeal subdomain Crenarchaeota [21]–[23].

The archaeal subdomain Euryarchaeota contains a large group of economically important organisms, including both the mesophilic and hyperthermophilic methane-producing organisms. The methane-producing genus *Methanosarcina* is known to harness all known substrates (including acetate, H_2_ and CO_2_, formate, methylamine and methanol) to produce methane, a greenhouse gas [24]. Recent reports also demonstrate that methanogens may be important in human health, especially from the nutritional standpoint [25], [26]. With the largest sequenced archaeal genome, *Methanosarcina acetivorans* C2A provides a fertile ground for studies on archaeal DNA metabolism. This organism has four putative topoisomerases (six genes) tentatively named MacTopoIIIα [NP_617416], MacTopoIIIβ [NP_616715], MacTopoVIA [NP_616519, NP_616520], MacTopoVIB [NP_616517, NP_616516] [24]. Based on sequence homology, MacTopoIIIα is classified as a type IA topoisomerase III protein. In the present report, we characterize MacTopoIIIα, as the first mesophilic archaeal TopoIII studied. The attributes shared with orthologs from the other domains and those potentially specific to Archaea are discussed.

## Results

### Expression and purification of MacTopoIIIα

**Figure 1:**
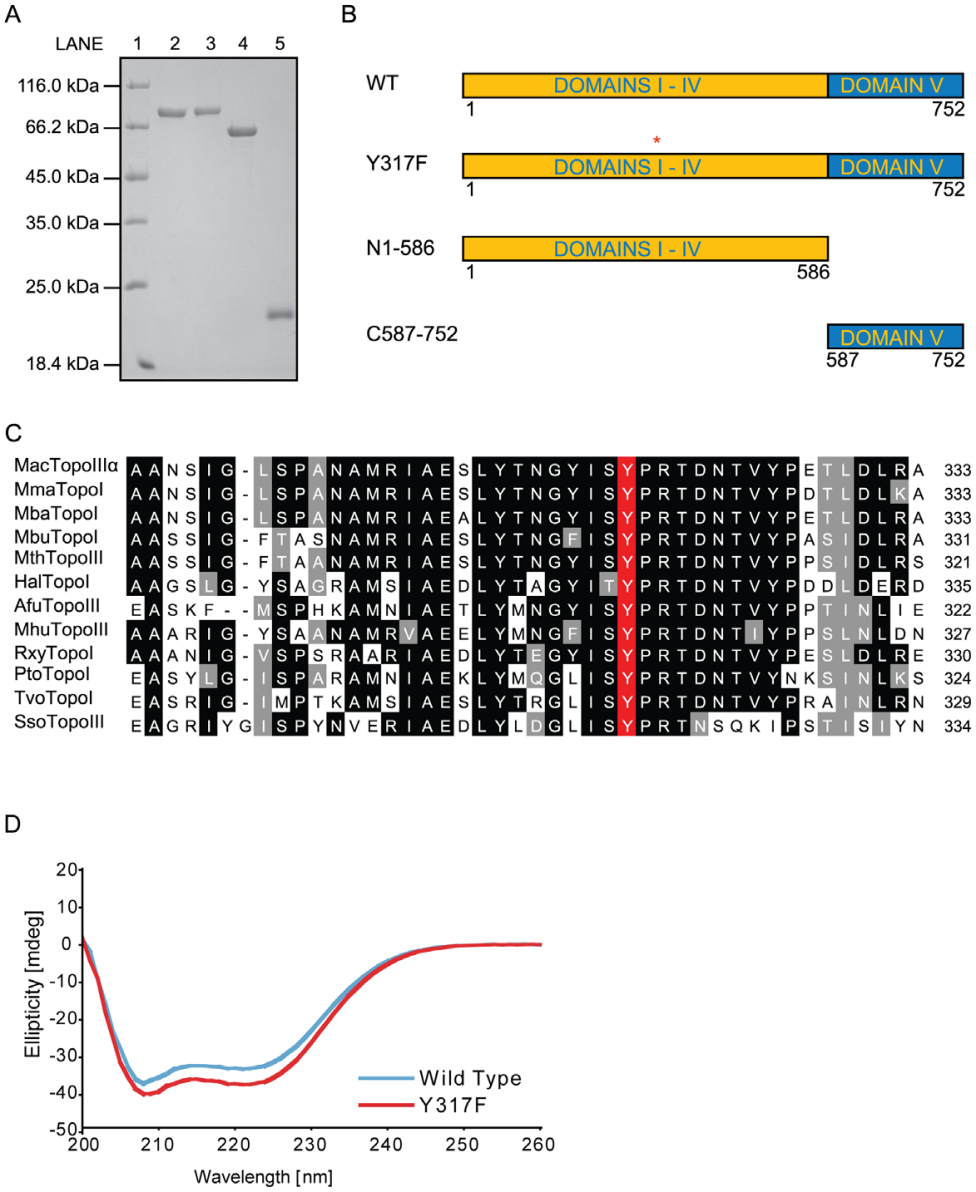
Purification and domain analysis of MacTopoIIIα. (A) SDS-PAGE of the purified recombinant proteins. Samples were loaded as follows: 1, Protein molecular mass markers (Fermentas); 2, MacTopoIIIα wild-type; 3, MacTopoIIIα Y317F; 4, MacTopoIIIα N1-586; 5, MacTopoIIIα C587–752. (B) Schematic representation of MacTopoIIIα wild-type, mutant (MacTopoIIIαY317F) and truncation (MacTopoIIIα C587–752 and MacTopoIIIα N1-586) proteins, showing the five canonical domains with Domains I-IV distinguished from the C-terminal Domain V. The red asterisk indicates location of the Y317F mutation. The domains are not drawn to scale. (C) Alignment of the active site of Archaeal TopoIII SubFamily II Topoisomerases with *Sulfolobus solfataricus* P2 TopoIII. MacTopoIIIα, *Methanosarcina acetivorans* C2A TopoIIIα [NP_617416]; MmaTopoI, *Methanosarcina mazei* TopoI [AAM32772]; MbaTopoI, *Methanosarcina barkeri* str. Fusaro TopoI [YP_305910]; MbuTopoI, *Methanococcoides burtonii* DSM 6242 TopoI [ABE51114]; MthTopoIII, *Methanosaeta thermophila* PT TopoIII [EAR48366]; HalTopoI, *Halobacterium* sp. NRC-1 TopoI [NP_444190]; AfuTopoIII, *Archaeoglobus fulgidus* DSM 4304 TopoIII [AAB89443]; MhuTopoIII, *Methanospirillum hungatei* JF-1 TopoIII [ABD40761]; RxyTopoI, *Rubrobacter xylanophilus* DSM 9941 TopoI [ABG04911]; PtoTopoI, *Picrophilus torridus* DSM 9790 TopoI [YP_023525]; TvoTopoI, *Thermoplasma volcanium* GSS1 TopoI [NP_110538]; SsoTopoIII, *Sulfolobus solfataricus* P2 TopoIII [NP_342400]. The GenBank accession numbers are in brackets. The conserved and similar amino acids are shaded black and gray, respectively. The putative catalytic residue is shaded red. (D) Circular Dichroism (CD) spectra of purified MacTopoIIIα wild-type and the Y317F mutant. Triplicate data sets were collected from samples at a concentration of 0.5 µg/µl in a buffer containing 50 mM Tris-HCl pH 7.5, 200 mM NaCl, 2 mM DTT. All data sets were normalized against baseline readings from buffer containing no protein.

In order to characterize MacTopoIIIα, the encoding gene was amplified, cloned, expressed, and purified from *E. coli* cells as a fusion protein with an N-terminal hexa-Histidine (6-His) tag. Purification of the recombinant protein was achieved through affinity chromatography, ion-exchange chromatography and size-exclusion chromatography. Based on the polypeptide sequence, the recombinant protein has an estimated molecular mass of ∼86 kDa. As expected, the highly purified MacTopoIIIα migrated between the 66 and 116 kDa molecular mass markers via a 12% sodium dodecyl sulfate-polyacrylamide gel electrophoresis (SDS-PAGE) according to the method of Laemmli (Fig. 1A, lane 2) [27].

### Catalytic properties of MacTopoIIIα

**Figure 2:**
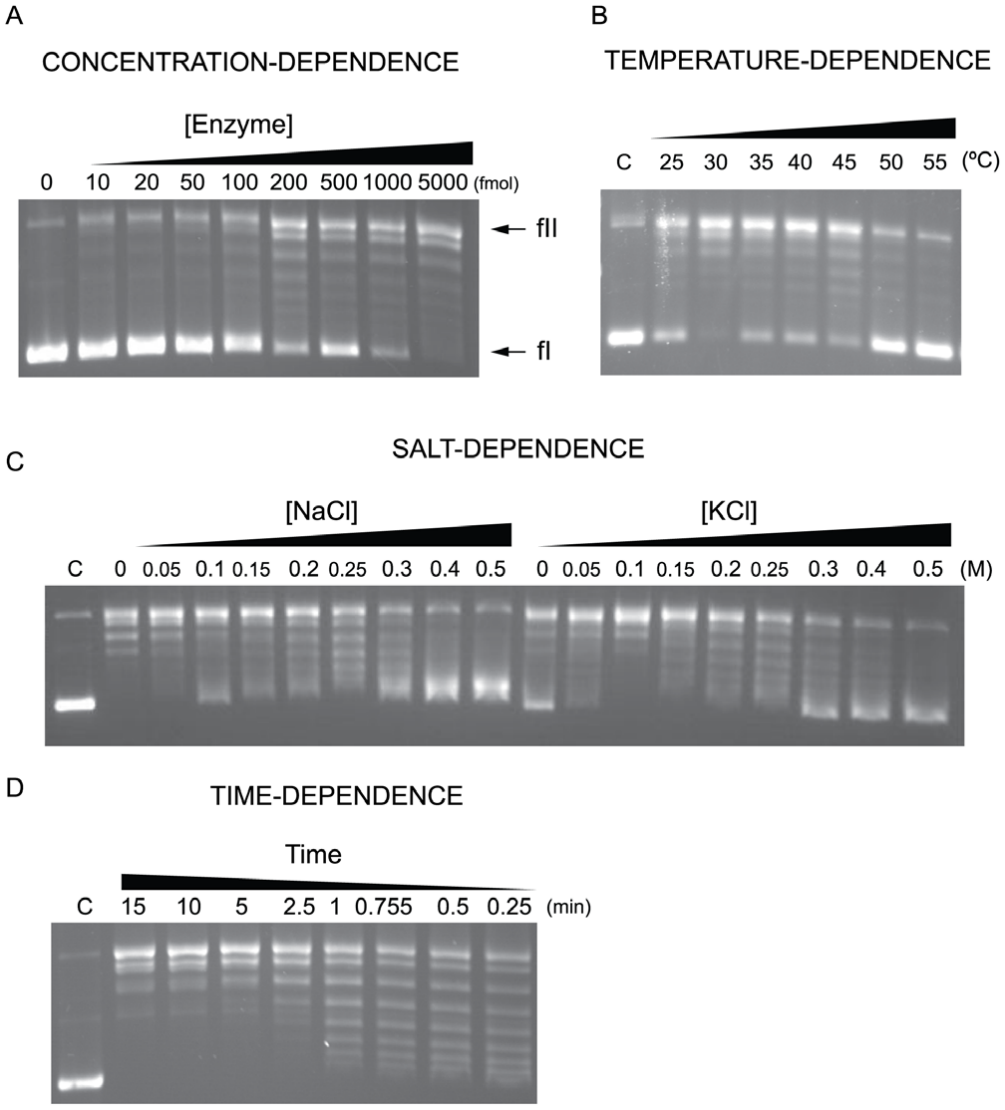
Characterization of activity based on protein concentration, salt and time. (A) Concentration dependent relaxation of negatively supercoiled pUC18 DNA by MacTopoIIIα wild-type. Standard reaction mixtures with 90 mM NaCl were incubated with 0.3 µg of pUC18 for 30 min at 37°C with 10 mM MgCl_2_. From left to right, lanes contain increasing amounts of enzyme as labeled. fI, form I; fII, form II. (B) Temperature dependent relaxation of negatively supercoiled pUC18 DNA by MacTopoIIIα wild-type. Standard reaction mixtures containing 5 pmol of enzyme were incubated with 0.3 µg of pUC18 for 30 min with 10 mM MgCl_2_. Lane C contains no divalent cation and was incubated at 37°C. The remaining lanes were incubated for 5 min at the indicated temperature prior to the addition of enzyme. (C) Salt dependent relaxation of negatively supercoiled pUC18 DNA by MacTopoIIIα wild-type. Standard reaction mixtures containing 5 pmol of enzyme were incubated with 0.3 µg of pUC18 for 30 min at 37°C with 10 mM MgCl_2_. Lane C contains 90 mM NaCl. From left to right, remaining lanes contain increasing amounts of NaCl or KCl as labeled. (D) Time dependent relaxation of negatively supercoiled pUC18 DNA by MacTopoIIIα wild-type. Standard reaction mixtures of enzyme were incubated with pUC18 at 37°C with 10 mM MgCl_2_. Lane C contains no divalent cation. Time points of the reaction were taken as indicated.

The putative topoisomerase was tested for the ability to relax negatively supercoiled pUC18 (300 ng). As little as 10 fmol of enzyme initiated relaxation upon addition to the reaction mixture (Fig. 2A). Whereas 200 fmol of enzyme showed modest activity, 5 pmol of enzyme elicited maximal activity under standard assay conditions. Analysis of aliquots of samples collected from the last step (gel filtration column) demonstrated similar activities in all fractions.

Maximal relaxation activity was observed around 30°C, and at temperatures of 50°C and above, no relaxation was observed (Fig. 2B). *M. acetivorans* C2A is an anaerobic mesophile that grows optimally at 35–40°C. Therefore, the optimal temperature for activity of MacTopoIIIα falls within the expected range of activity [28]. Both Na+ and Mg2+ are required for growth of this archaeon, and the optimal concentration of NaCl required was 200 mM. In the present study, at NaCl concentrations of 400 mM or higher, the activity of MacTopoIIIα was almost not observable (Fig. 2C). A similar observation was made for the effect of KCl concentration on MacTopoIIIα activity. Initiation of activity by the enzyme was observable as early as 15 seconds after addition of MacTopoIIIα to the reaction mixture, and maximal activity was observed within 5 min (Fig. 2D).

### The effects of different divalent cations on MacTopoIIIα activity

**Figure 3:**
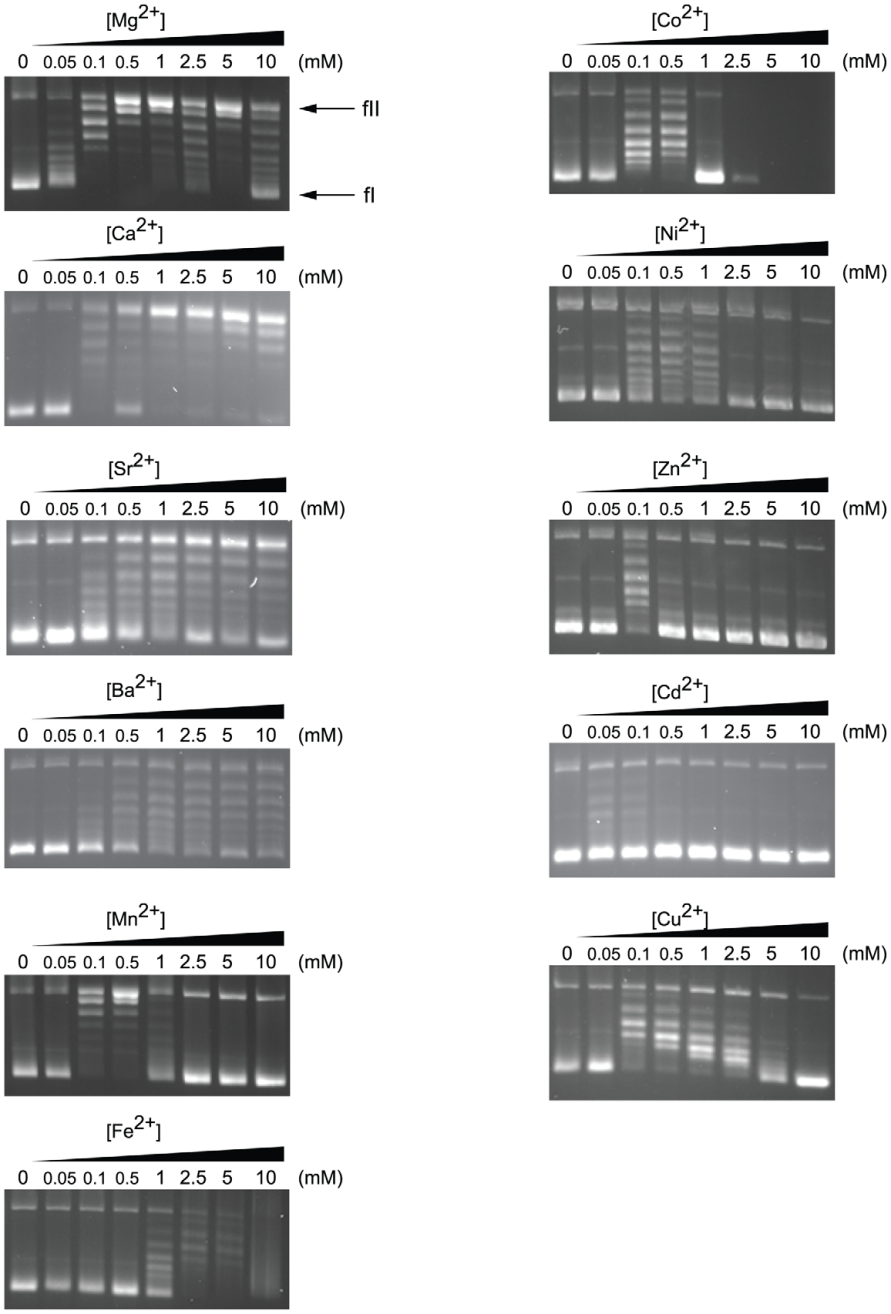
Cation-dependent DNA relaxation activity profiles of MacTopoIIIα wild-type. Relaxation of negatively supercoiled pUC18 DNA by MacTopoIIIα wild-type. Standard reaction mixtures with 90 mM NaCl containing 5 pmol of enzyme were incubated with 0.3 µg of pUC18 for 30 min at 37°C with the corresponding divalent cation. Lanes 1–8 contain increasing amounts of the indicated divalent cation: Lane 1, 0 µM; Lane 2, 50 µM; Lane 3, 100 µM; Lane 4, 500 µM; Lane 5, 1 mM; Lane 6, 2.5 mM; Lane 7, 5 mM; Lane 8, 10 mM. fI, form I; fII, form II.

Topoisomerase activity was assessed as a function of divalent cation concentration in a semi-logarithmic range of concentrations from 50 µM to 10 mM (Fig. 3). We observed that each divalent cation elicited a unique activity profile. As in previous studies on topoisomerases, Mg^2+^ was able to elicit maximal activity at concentrations greater than or equal to 1 mM. Ca^2+^ was the only other cation that maintained a profile comparable to Mg^2+^, with concentrations greater than 1 mM showing near maximal relaxation. In fact, relaxation activity of MacTopoIIIα at concentrations above 1 mM appeared to be higher for Ca^2+^ compared with Mg^2+^. Ba^2+^ and Sr^2+^ profiles were similar, but were not able to relax the supercoiled substrate as effectively as Mg^2+^ and Ca^2+^. The ability of these Group II alkaline metals to elicit DNA relaxation activity at low cation concentration was, therefore, as follows: Mg^2+^ > Ca^2+^ >> Ba^2+^ > Sr^2+^.

In the presence of Zn2+, activity was observed in a narrow window of 100 µM. Zn2+ concentrations of 50 µM or less and 500 µM or more elicited no activity even when the reaction was performed in a 1∶1 enzyme:DNA molar ratio (data not shown). To a lesser extent, Cd2+ was also able to elicit activity within a similar narrow range. Relaxation activity was observed at 0.1 mM and 0.5 mM concentrations of Co2+, and at a concentration of 1 mM or higher, either no activity was detectable or divalent cation-catalyzed DNA degradation was observed. Fe2+ required a concentration of 1 mM or higher for activity. However, DNA degradation was also significant at concentrations above 1 mM. These results are consistent with metal catalyzed DNA degradation seen with certain transition metals that are capable of promoting the Haber-Weiss reaction [29], [30]. Relaxation activity in the presence of Mn2+ or Ni2+ or Cu2+ was also elicited under a narrow range (100 µM – 1 mM).

### Classification of MacTopoIIIα

To determine the class of MacTopoIIIα in the topoisomerase family, its response to known topoisomerase inhibitors, with different mechanisms of action, was investigated. Table 1 shows a summary of the inhibitors tested. Ethidium bromide (EtBr) and M13 ssDNA inhibited activity. The type I prokaryotic inhibitor spermidine and the type I eukaryotic inhibitor camptothecin were unable to inhibit the activity of MacTopoIIIα. Furthermore, the type II inhibitors (etoposide, nalidixic acid and novobiocin) also had no effect on the relaxation activity of MacTopoIIIα. Relaxation activity was, however, inhibited by high concentrations of KCl and NaCl (>400 mM). Based also on preliminary analytical gel filtration experiments, MacTopoIIIα was estimated to exist as a monomer in solution, which is consistent with the oligomerization state of type 1A topoisomerases [3].

**Table 1:** Summary of inhibition assays conducted with MacTopoIIIα wild-type.

Chemical	Inhibition
Ethidium Bromide	Yes
M13 ssDNA	Yes
Spermidine	No
Camptothecin	No
Etoposide	No
Nalidixic acid	No
Novobiocin	No
High [KCl]	Yes
High [NaCl]	Yes

Three independent trials were conducted to determine the ability of different agents to inhibit DNA relaxation activity at increasing concentrations.

### Catalytic Tyrosine of MacTopoIIIα is Y317

**Figure 4:**
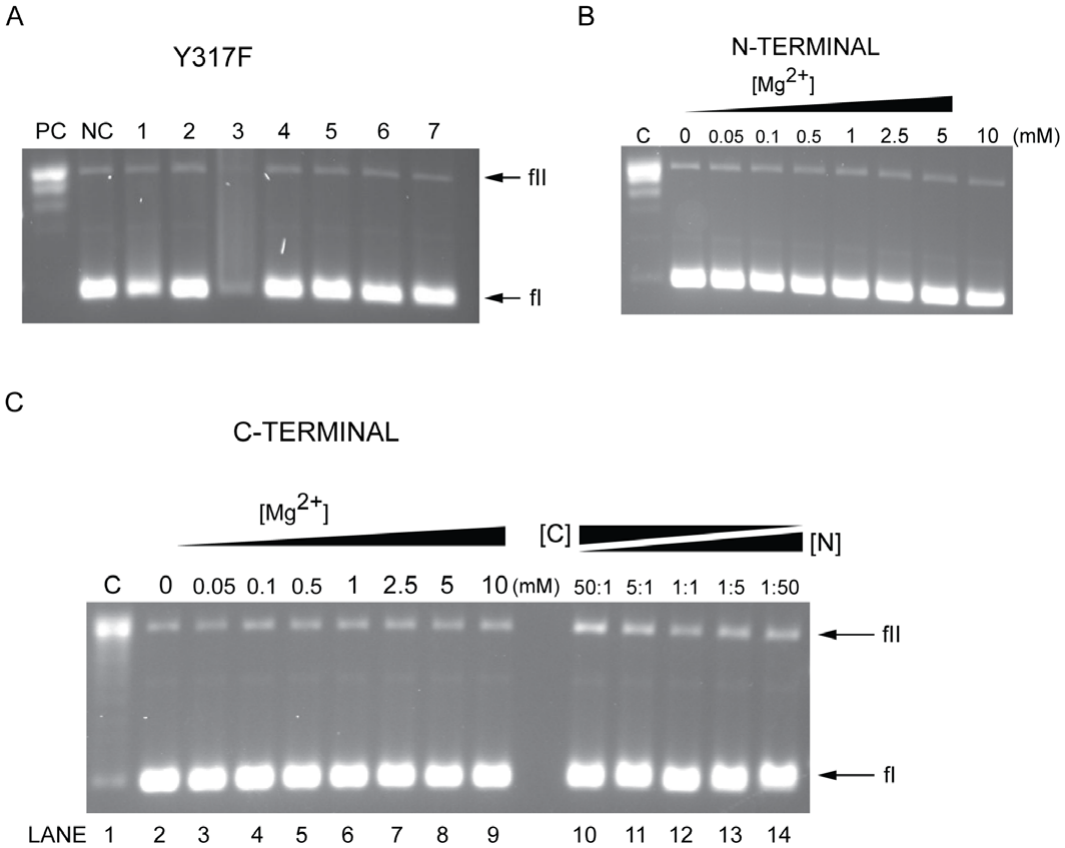
Mutant and truncation DNA relaxation assays. (A) Standard reaction mixtures with 90 mM NaCl were incubated in the presence of 5 pmol of MacTopoIIIα Y317F with 0.3 µg of pUC18 for 30 min at 37°C unless stated otherwise. Lane PC contains 10 mM MgCl_2_ and 5 pmol of wild-type enzyme only. Lane NC contains MacTopoIIIα Y317F only. Lane 1, 500 µM CoCl_2_; Lane 2, 100 µM CuCl_2_; Lane 3, 2 mM FeCl_2_; Lane 4, 10 mM MgCl_2_; Lane 5, 50 µM MnCl_2_; Lane 6, 500 µM NiCl_2_; Lane 7, 100 µM ZnCl_2_. fI, form I; fII, form II. (B) Relaxation of negatively supercoiled pUC18 DNA by MacTopoIIIα N1-586. Standard reaction mixtures with 90 mM NaCl containing 5 pmol of enzyme were incubated with 0.3 µg of pUC18 for 30 min at 37°C. Lane 1 contains 10 mM MgCl_2_ and 5 pmol of MacTopoIIIα wild-type only. Lanes 2–9 contain increasing amounts of MgCl_2_: Lane 2, 0 µM; Lane 3, 50 µM; Lane 4, 100 µM; Lane 5, 500 µM; Lane 6, 1 mM; Lane 7, 2.5 mM; Lane 8, 5 mM; Lane 9, 10 mM. fI, form I; fII, form II. (C) Relaxation of negatively supercoiled pUC18 DNA by MacTopoIIIα C587–752. Standard reaction mixtures with 90 mM NaCl containing 5 pmol of enzyme were incubated with 0.3 µg of pUC18 for 30 min at 37°C. Lane 1 contains 10 mM MgCl_2_ and 5 pmol of MacTopoIIIα wild-type only. Lanes 2–9 contain increasing amounts of MgCl_2_: Lane 2, 0 µM; Lane 3, 50 µM; Lane 4, 100 µM; Lane 5, 500 µM; Lane 6, 1 mM; Lane 7, 2.5 mM; Lane 8, 5 mM; Lane 9, 10 mM. Lanes 10–14 contain varying ratios of MacTopoIIIα C587–752 to MacTopoIIIα N1-586 ranging from 50∶1, to 5∶1 to 1∶1 to 1∶5 to 1∶50, respectively. fI, form I; fII, form II.

A sequence alignment using *Sulfolobus solfataricus* topoisomerase III (SsoTopoIII) and the entire archaeal TopoIII subfamily II (of which MacTopoIIIα is a member) was created to provide a hint at the potential catalytic tyrosine (Fig. 1C), because SsoTopoIII is the only archaeal TopoIII with a characterized active site tyrosine [9], [22]. The alignment suggested Tyr317 as the potential active site residue of interest. Site-directed mutagenesis was used to create a Y317F mutant. Size exclusion chromatography of MacTopoIIIα Y317F yielded similar elution volume to that of the wild-type protein, suggesting that the mutant also exists as a monomer in solution (data not shown). The purified wild-type and mutant proteins (Fig. 1A, lanes 2 and 3) were subjected to circular dichroism scan to determine whether the mutation grossly impacted the structure of MacTopoIIIα Y317F (Fig. 1C). No gross differences between the structures could be discerned, suggesting that the secondary structures of both enzymes are comparable, outside of the point mutation. By subjecting the purified mutant protein to the relaxation reaction, it was revealed that MacTopoIIIα Y317F was unable to relax the negatively supercoiled substrate under the same conditions where the wild-type protein demonstrated activity (Fig. 4A). The products seen in Lane 5 are likely to be due to iron catalyzed DNA degradation via the Haber-Weiss reaction (refer to Fig. 3, last lane under Fe2+).

### Truncation mutants

Two truncations of MacTopoIIIα were constructed based on sequence alignment with the 67 kDa fragment of EcoTopoI [31]. Based on this sequence alignment, the MacTopoIIIα sequence was spliced at a position between two small aliphatic residues (A586 and I587) where no secondary structure was apparent based on secondary structure prediction (PredictProtein: http://www.predictprotein.org/). We constructed two fragments, N1-586 and C587–752, containing the N-terminal 586 amino acid residues and the C-terminal 166 amino acid residues, respectively. Both truncations were cloned, overexpressed and highly purified. Neither the N1-586 nor the C587–752 fragment was able to relax the same substrate as the wild-type under standard assay conditions (Fig. 4B and 4C). To determine whether relaxation activity could be reconstituted by mixing the two fragments, they were incubated briefly prior to addition to the reaction mixture at varying ratios (50∶1, 5∶1 and 1∶1). However, no relaxation activity was observed (Fig. 4C). To determine whether higher concentrations of the two fragments will elicit topoisomerase activity, we examined concentrations up to five times the maximum concentration used for the wild-type protein; however, no relaxation of the substrate was observed either by the individual polypeptides or their mixture (Fig. S1). The polypeptides, especially the C587-752 polypeptide, demonstrated DNA binding activity at high concentrations. Further experiments will be designed to characterize this property of the split MacTopoIIIα.

### Zinc Content Measurements

**Figure 5:**
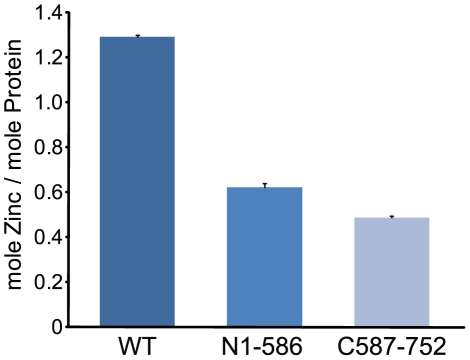
ICP-MS analysis of MacTopoIIIα wild-type and its truncations. Purified recombinant MacTopoIIIα wild-type and truncation (MacTopoIIIαN1-586 and MacTopoIIIαC587–752) proteins were submitted for zinc content analysis. The error bars represent standard deviations for two independent measurements.

In an effort to assess the Zn^2+^ binding ability of this enzyme, samples of wild-type and the two truncation mutants were subjected to ICP-MS analysis. The wild-type enzyme was found to contain 1.29±0.01 mol of Zn^2+^. The MacTopoIIIα N1-586 truncation was found to contain 0.62±0.02 mol, and the C587–752 truncation mutant was found to contain 0.49±0.01 mol of Zn^2+^ (Fig. 5).

## Discussion

### MacTopoIIIα is promiscuous

A fundamental property of type IA topoisomerases is the absolute requirement of Mg^2+^ for the large conformational change required during DNA relaxation, but other divalent cations can substitute [5], [32]. Recent publications have proposed a two metal mechanism for type IA and type II topoisomerases wherein one may bind acidic residues within the TOPRIM motif causing the necessary large conformational changes while the other may play a transient role in stabilizing the transition state in preparation for attack on the scissile phosphate [33], [34]. Fluorescence and ICP-MS measurements on *E. coli* TopoI (EcoTopoI) reveal that one Mg^2+^ plays a role influencing the overall structure of the enzyme, while a second Mg^2+^ is required to achieve full catalytic activity [35]. Mutation of three acidic residues of EcoTopoI within the TOPRIM domain only partially inhibited activity [36]. But even when crystals were soaked in MgCl_2_, no Mg^2+^ was detected in the crystal structures of EcoTopoIII whether the wild-type was covalently linked or whether the wild-type or mutant were primed for attack at a scissile bond of ssDNA in a manner akin to *S. cerevisiae* TopoII (SceTopoII) [37], [38]. The data in total suggests that two transient divalent cations are required for maximal activity with one divalent cation binding in order to prime the enzymatic structure for large conformational changes while the second cation primes the phosphate linkage of interest for attack before immediate removal from the active site.

And while the role of Mg^2+^ is usually well examined, it is commonplace for other divalent cations to receive less scrutiny (Table 2). As one example, EcoTopoI DNA relaxation activity was investigated in the presence of 0–10 mM Mg^2+^ while Ca^2+^ , Co^2+^, Mn^2+^ and Zn^2+^ were investigated at 2 mM only [39]. In another study, while the activity of human topoisomerase IIIα (hTopoIIIα) was again tested in the presence of a range of MgCl_2_ concentrations, hTopoIIIα was deemed inactive in the presence of other divalent cations based on one concentration (5 mM) [40]. We demonstrate for the first time that MacTopoIIIα is promiscuous with regard to divalent cation (Ba^2+^, Ca^2+^, Cd^2+^, Co^2+^, Cu^2+^, Fe^2+^, Mg^2+^, Mn^2+^, Ni^2+^, Zn^2+^) and that each elicits a unique activity profile. Unlike results with other topoisomerases, we observe DNA relaxation activity in the presence of Zn^2+^ albeit under a narrow range [22], [32]. These results are not plasmid-specific as confirmed in the presence of 300 ng of either pUC18 or pGEM-T (data not shown). While further studies are needed to determine if all divalent cations may substitute in the mechanism for all topoisomerases, our data clearly demonstrates that there is a need to assess bivalent metal ion profiles and not simply examine activity at a singular concentration. As a result, it is possible that different divalent cations not previously reported to elicit activity for any given topoisomerase indeed do elicit activity, although it may be under a narrow range of concentrations. It also remains to be seen whether these divalent cation profiles can act as a tool to distinguish topoisomerases or whether this activity is specific to Archaea or to this particular archaeon in the present study.

**Table 2:** Summary of divalent cation studies.

Enzyme	Mg^**2+**^	Ca^**2+**^	Sr^**2+**^	Ba^**2+**^	Mn^**2+**^	Fe^**2+**^	Fe^3+^	Fe^4+^	Co^**2+**^	Ni^**2+**^	Cu^**2+**^	Zn^**2+**^	Cd^**2+**^	Ref
MacTopoIIIα	Y	Y	M	M	Y	Y	-	-	Y	M	Y	M	M	This paper
DmeTopoIIIβ	Y	Y	-	-	Y	-	-	-	N	-	N^S^	N^S^	-	[53]
EcoTopoI	Y	Y^D^	-	-	N^D^	-	-	-	L^D^	-	-	L^D^	-	[32]
FisTopoI	Y	N^D^	-	-	N^D^	-	-	-	-	-	N^D^	-	N^D^	[45]
HsaTopoIIIα	Y	-	-	-	N	-	-	-	N	N	N	N^S^	-	[40]
MetTopI	Y	M	-	-	Y	Y	Y	Y	-	-	Y	N	-	[49]
NtaTopoI	Y	Y^D^	-	-	M^D^	-	-	-		-	N^D^	N^D^	-	[54]
RcaTopoI	Y	Y	-	-	Y	-	-	-	-	-	N	N	-	[55]
SauTopoI	Y	Y	-	-	Y	-	-	-	-	-	N	N	-	[46]
SsoTopoIII	Y	M	-	-	N	-	-	-	N	N	N	N	-	[22]

A literature review was conducted on previously published studies where divalent cations other than Mg^**2+**^ were utilized in type IA topoisomerase DNA relaxation assays. The findings are summarized herein. *Methanosarcina acetivorans* C2A, Mac; *Drosophila melanogaster*, Dme; *Escherichia coli*, Eco; *Fervidobacterium islandicum*, Fis; *Homo sapiens*, Hsa; *Methylophaga* sp. strain 3, Met; *Nicotiana tabacum*, Nta; *Rhodobacter capsulatus*, Rca; *Staphylococcus aureus*, Sau; *Sulfolobus solfataricus*, Sso; ^D^, data not shown; ^S^, M^**2+**^SO_4_ used in place of M^**2+**^Cl_2_; Y, high activity; M, moderate activity; L, low activity; N, no activity.

### MacTopoIIIα is a type IA topoisomerase

Nalidixic acid, etoposide and novobiocin are specific inhibitors of type II topoisomerases with different mechanisms of action [10]. Camptothecin is a known eukaryotic type I inhibitor that stabilizes the binary protein-DNA complex intermediate thereby impeding DNA relaxation [41]. None of these inhibitors were able to inhibit the DNA relaxation activity of MacTopoIIIα. Ethidium bromide induces positive supercoiling [42]. Type IA topoisomerases have a preference for ssDNA and preferentially relax negatively supercoiled DNA. Ethidium bromide and M13 ssDNA were each able to inhibit activity. Thus, the sequence homology in combination with the results from the inhibitor studies clearly demonstrates that MacTopoIIIα is a type IA topoisomerase.

Spermidine had no effect on MacTopoIIIα at concentrations up to 100 mM. Spermidine was initially posited as a prokaryotic polyamine topoisomerase inhibitor given its inhibitory effect on both EcoTopoI and EcoTopoIII at physiological concentrations [43], [44]. However, the data actually suggests that it is a case by case scenario within the domain Bacteria and the only archaeal TopoIII tested to-date, SsoTopoIII, demonstrated no susceptibility [22], [45]–[49]. The data reveal that there is no definite trend for polyamine susceptibility amongst bacterial type IA topoisomerases and no demonstrated effect to-date in the domain Archaea.

### Catalytic tyrosine is located at Y317

We assayed the Y317F mutant of MacTopoIIIα for activity under conditions that were demonstrated to elicit relaxation activity for wild-type. After analyzing the CD spectra of MacTopoIIIα Y317F to ensure that there were no gross structural differences compared to the wild-type, we demonstrated that MacTopoIIIα Y317F was inactive under conditions where wild-type had activity. This result confirmed our prediction that Tyr317 is indeed essential in the mechanism of DNA relaxation by MacTopoIIIα.

### N-terminal Zn^2+^-binding activity

An open question about TopoI and TopoIII is the role that the C-terminal (Domain V) plays in the context of Zn^2+^-binding. While lacking the C-terminal (Domain V), the EcoTopoI fragment (Domains I–IV) retained the ability to cleave oligonucleotides, but was not able to relax negatively supercoiled DNA [31]. With three Zn^2+^-binding sites in Domain V (the domain absent in the original crystal structure), methyl methanethiosulfonate (MMTS)-treated EcoTopoI abolished Zn^2+^-binding and rendered the enzyme incapable of catalyzing DNA relaxation [32]. Meanwhile, a double mutant (Cys559A/Cys561A) or truncation that abolished Zn^2+^-binding in *Thermotoga maritima* TopoI (TmTopoI) only minimally affected DNA relaxation activity [4]. No TopoI crystal structures have been identified with Zn^2+^ positioned even when it was detected in the mother crystal liquor [50]. The story is further confounded by the fact that there are several bacterial type IA topoisomerases that contain no homologous Zn^2+^-binding motif or Zn^2+^-binding ability [37], [48], [51]. A reasonable conclusion from the current data available is that type IA topoisomerase Zn^2+^-binding motifs, when present in a given bacterial organism, play a structural role that may or may not be essential for DNA relaxation activity.

Given that MacTopoIIIα contains a C-terminal Cys-X_2_-Cys-X_17_-Cys-X_5_-Cys motif and that there have not been any studies to date examining the role of Zn^2+^-binding in archaeal TopoIII, we set out to determine whether MacTopoIIIα contains Zn^2+^ in order to delineate any potential differences with the well-characterized bacterial topoisomerases. The results demonstrated that MacTopoIIIα binds to approximately one mole of Zn^2+^ per mole of protein. We then constructed, expressed and purified N-terminal and C-terminal fragments to assess the Zn^2+^-binding ability of each segment. To our surprise, both the C-terminal fragment and the N-terminal fragment demonstrated the ability to chelate approximately a half mole of Zn^2+^. To our knowledge, this is the first report of an N-terminal fragment of a TopoIII or TopoI having this ability to coordinate Zn^2+^. It is likely that residues in the two fragments coordinate Zn^2+^ in the wild-type. Mutational studies are currently underway to investigate the Zn^2+^-binding role that any of the eight His residues in the N-terminal and eleven Cys/His residues in the C-terminal may play.

In this report, MacTopoIIIα, a mesophilic archaeal TopoIII, was biochemically characterized in the context of well-established properties of eukaryotic and bacterial type IA topoisomerases. We demonstrate that MacTopoIIIα is a monomer in solution, binds Zn^2+^ and exhibits susceptibility to inhibitors in a manner similar to other type IA topoisomerases. MacTopoIIIα is a distributive topoisomerase that has a high affinity for ssDNA and is inhibited by high salt concentrations. Properties that may be unique to this enzyme are the ability of all divalent cations tested to elicit DNA relaxation activity and the ability of this enzyme to bind Zn^2+^ via the N- and C-terminal. Further studies are needed in the domain Archaea and beyond to determine if these attributes are domain or organism specific.

## Materials and Methods

Cloning and expression of *M. acetivorans* C2A TopoIIIα (MacTopoIIIα) gene

The gene for the putative topoisomerase, MacTopoIIIα (NP_617416), was amplified from the *M. acetivorans* C2A genomic DNA using PCR primers containing restriction sites for NdeI and XhoI in the forward (5′-CATATGCACCTTATCGTAACGGAAAAAAATATA-3′) and reverse (5′-CTCGAGCTATAGGTCCTCAACTATGCCGCCGTTACA-3′) primers, respectively. The size of the PCR product was verified through agarose gel electrophoresis and cloned into pGEM-T, a TA-cloning vector (Qiagen). The recombinant plasmid was purified using a Qiagen gel extraction kit, and after confirming the integrity of the sequence of the DNA insert, the MacTopoIIIα gene was transferred into a modified pET28a expression vector. The pET28a vector has its kanamycin resistance gene replaced with an ampicillin resistance gene [52]. Therefore, the ligated products were transformed into *E. coli* JM109 cells and transformants were selected on lysogeny broth (LB) plates supplemented with ampicillin at a final concentration of 100 µg/mL. The plates were incubated overnight at 37°C, and a single colony was picked and cultured in 10 mL LB containing the same antibiotic and grown at 37°C for 8 hours. The plasmid was extracted from the *E. coli* culture and examined for the presence of the MacTopoIIIα gene through DNA sequencing. The recombinant plasmid was named pET28a/MacTopoIIIα. All DNA sequencing in the present report were carried out at the W. M. Keck Center for Comparative and Functional Genomics (University of Illinois at Urbana-Champaign).

The recombinant plasmid pET28/MacTopoIIIα was used in transforming *E. coli* BL21 codonplus RIPL cells (Stratagene) and plated on LB plates supplemented with ampicillin (100 µg/mL) and chloramphenicol (50 µg/mL). A transformant was cultured at 37°C in 10 ml LB medium supplemented with both antibiotics at the same concentrations stated above until the optical density (O.D.) at 600 nm reached 0.3. The expression of the MacTopoIIIα gene was then induced by adding isopropyl β-D-thiogalactopyranoside (IPTG) at a final concentration of 1 mM. The temperature was decrease to 16°C and cell culturing was continued for 12–16 hours. The recombinant *E. coli* cells were then collected through centrifugation at 6500 rpm for 10 minutes.

### Site-directed mutagenesis

Site-directed mutagenesis was carried out using the Quikchange site-directed mutagenesis kit (Stratagene) according to the instructions of the manufacturer. The PCR primer (5′-AACGGGTATATATCTTTTCCCAGGACCGACAAT-3′) was utilized to convert MacTopoIIIα Tyr317 into Phe317. Mutagenized plasmid was transformed into JM109 cells after digestion of the parental plasmid with DpnI. The *E. coli* transformants were selected on LB agar plates supplemented with ampicillin [100 µg/mL] overnight at 37°C. Plasmids were extracted from individual colonies after growth in liquid LB cultures supplemented with ampicillin [100 µg/mL]. Plasmids were sequenced as described above, and the desired plasmid containing the Y317F mutation was selected for gene expression as described above for the wild-type gene.

### Truncation mutagenesis

A truncated gene containing the DNA sequence for the N-terminal 586 amino acids (N1-586) was generated using an iProof™ High-Fidelity PCR kit with a forward primer (5′-CATATGCACCTTATCGTAACGGAAAAAAATATA-3′) and reverse primer (5′-CTCGAGCTATGCCTGCAGGGACTCTATGATTTTGTC-3′) according to the manufacturer's instructions. A truncated gene containing the DNA sequence for the C-terminal 166 amino acids (C587–752) was generated in the same manner with the forward primer (5′-CATATGGGTCTCAGGGAAGACAAGATCATAGGCAAC-3′) and the reverse primer (5′-CTCGAGCTATAGGTCCTCAACTATGCCGCCGTTACA-3′). The size of the PCR product was verified through agarose gel electrophoresis and cloned into the pGEM-T vector (Qiagen). Plasmids were transformed into JM109 cells after ligation. The *E. coli* transformants were selected on LB agar plates supplemented with ampicillin [100 µg/mL] overnight at 37°C. Plasmids were extracted from individual colonies after growth in liquid cultures supplemented with ampicillin [100 µg/mL]. Plasmids were sequenced as described above, and the correct inserts of N1-586 and C587–752 were transferred to the modified pET28a for expression as described above.

### Purification of MacTopoIIIα and mutant proteins

For MacTopoIIIα wild-type protein and the Y317F mutant, the harvested recombinant *E. coli* cells were resuspended in Buffer A (50 mM Sodium phosphate, pH 7.0, 300 mM NaCl), and the cell contents were released by a French pressure cell (American Instruments Co). The cell debris was removed through centrifugation at 9500 rpm for 15 min at 4°C. The supernatant was subsequently filtered using a 0.22 µm filter and applied to a His-Trap^TM^ HP column (GE Healthcare) pre-equilibrated with 90% buffer A and 10% buffer B (50 mM Sodium phosphate, pH 7.0, 300 mM NaCl, 500 mM imidazole), and eluted using a step-wise gradient of Buffer B.

Fractions containing the protein of interest were pooled and dialyzed against buffer A (50 mM Sodium phosphate, pH 7.0, 300 mM NaCl) overnight at 4°C. Samples were applied to a HiTrap^TM^ SP column (GE Healthcare) pre-equilibrated with buffer A, and to elute the bound protein, a linear gradient using 100% buffer C (50 mM Sodium phosphate, pH 7.0, 1 M NaCl) was utilized. Concentrated fractions of each protein were directly applied to a size exclusion column (Superdex HR 200 HR 10/30 column) pre-equilibrated with buffer GF (50 mM sodium phosphate, 150 mM sodium chloride, pH 7.0), and the chromatography was developed with the same buffer. Highly purified proteins were dialyzed against a storage buffer (50 mM Tris-HCl pH 7.5, 200 mM NaCl, 2 mM DTT and 50% Glycerol) and stored at −80°C until used.

In the case of MacTopoIIIα C587–752 truncated mutant, the harvested recombinant *E. coli* cells were re-suspended in Buffer A, and the cell contents were released by a French pressure cell (American Instruments Co). The cell debris was removed through centrifugation at 9500 rpm for 15 min at 4°C. The supernatant was subsequently filtered using a 0.22 µm filter and applied to a His-Trap^TM^ HP column (GE Healthcare) pre-equilibrated with 70% buffer A and 30% buffer B (50 mM Sodium phosphate, pH 7.0, 300 mM NaCl, 500 mM imidazole), and eluted using a linear gradient of buffer B.

The protein fractions from the HisTrap^TM^ HP column were pooled and dialyzed against buffer A (50 mM Sodium phosphate, pH 7.0, 300 mM NaCl) overnight at 4°C. Samples were applied to a HiTrap^TM^ SP column (GE Healthcare) pre-equilibrated with buffer A and eluted with a linear gradient of 100% buffer C. Highly purified fractions were dialyzed against the storage buffer and stored at −80°C until used.

In the case of MacTopoIIIα N1-586 truncated mutant, the harvested recombinant *E. coli* cells were re-suspended in Buffer A, and the cell contents were released by a French pressure cell (American Instruments Co). The cell debris was removed through centrifugation at 9500 rpm for 15 min at 4°C. The supernatant was subsequently filtered using a 0.22 µm filter and applied to a His-Trap^TM^ HP column (GE Healthcare) pre-equilibrated with 75% buffer A and 25% buffer B (50 mM Sodium phosphate, pH 7.0, 300 mM NaCl, 500 mM imidazole), and eluted using a linear gradient of buffer B.

The fractions of the MacTopoIIIα N1-586 truncated mutant from the His-Trap column were pooled and dialyzed against buffer A overnight at 4°C. Samples were then applied to a HiTrap^TM^ heparin HP column (GE Healthcare) pre-equilibrated with 90% buffer A and 10% buffer C. The MacTopoIIIα N1-586 truncated mutant was eluted with a step gradient using 75% buffer A and 25% buffer C. Highly purified fractions were dialyzed against the storage buffer and stored at −80°C until used. Aliquots of eluted fractions from all chromatographies were examined through SDS-PAGE.

### Preparation of DNA substrates for topoisomerase assays

*E. coli* JM109 cells were heat shocked to uptake the pUC18 plasmid, and transformants were cultured in ampicillin supplemented (100 µg/mL) LB medium to amplify the plasmid. The pUC18 plasmid was purified from cell pellets with a commercial kit (Qiagen), and electrophoresed on a 1.5% agarose gel containing 1X TBE (89 mM Tris, 89 mM Boric acid, 2 mM EDTA). Subsequently, the gel was stained with ethidium bromide and imaged with a UV illuminator. The plasmid DNA migrating the fastest was excised and purified utilizing a gel extraction kit (Qiagen) according to the manufacturer's specifications. The purified negatively supercoiled DNA was verified using a 2-Dimensional gel with EcoRI linearized pUC18 and ethidium bromide treated pUC18 as standards. The M13 ssDNA was from a commercial source (New England Biolabs).

### DNA relaxation assay

Unless otherwise stated, the method is the same as previously described [22]. Briefly, reaction volumes contained 300 ng negatively supercoiled pUC18 DNA in the presence of 5 pmol of MacTopoIIIα wild-type (or its mutants) in 50 mM Tris-HCl, pH 8.8, 1 mM DTT, 0.1 mM EDTA, 90 mM NaCl, 30 µg of BSA/ml, and 12% (vol/vol) ethylene glycol with the indicated divalent cation. After 30 min incubation at 37°C (unless indicated otherwise), reactions were terminated by adding 4 µL of DNA loading dye (25% Glycerol, 0.2% Bromophenol Blue, 50 mM EDTA) and the products were resolved with 1.5% agarose gel in 0.5X TPE (44.6 mM Tris, 0.13% Phosphoric acid, 1 mM EDTA) buffer. DNA bands were visualized through ethidium bromide staining and imaging with a UV illuminator.

The levels of negatively scDNA that remained or were relaxed (partially and completely) were determined by densitometry using AlphaEaseFC^TM^ software. For each divalent ion, the values for a given lane were normalized to that obtained without any divalent cation present (Fig 3, Lane 1). Activity for each ion was assessed according to the maximal percentage of substrate that was relaxed (partially and completely) relative to the percentage of negatively scDNA when no ion was present. High activity (Y) was utilized to denote greater than or equal to 95% activity while medium activity (M) denotes activity falling below 95%.

### Determination of zinc content by ICP-MS

The zinc content in purified wild-type MacTopoIIIα and variants was determined at the University of Illinois Microanalysis Laboratory using the SCIEX ELAN DRCe ICP-MS (PerkinElmer Life Sciences). Briefly, two aliquots of purified protein, 1 mL each, at a concentration of approximately 0.5 mg/mL, were digested in nitric acid. The resulting solutions were diluted to 25 mL and analyzed. Two isotopes of zinc, ^64^Zn and ^66^Zn, were analyzed with gallium as the internal standard.

### Estimation of subunit organization by gel filtration

Since MacTopoIIIα was expressed with an N-terminal 6-His tag, the protein eluted from the HiTrap^TM^ SP column was incubated with the protease thrombin (1 unit/mg/mL) during dialysis in buffer GF (50 mM sodium phosphate, 150 mM sodium chloride, pH 7.0) in order to remove the tag. The dialyzate was loaded onto a Superdex 200 HR 10/300 gel filtration column pre-equilibrated with buffer GF. The chromatography was developed with the same buffer at a flow rate of 0.5 ml/min and fractions of 0.5 ml were collected and analyzed by SDS-PAGE. To generate a standard curve, the column was calibrated by analyzing a set of gel filtration standards (thyroglobulin, 669 kDa; bovine γ-globulin, 158 kDa; chicken ovalbumin, 44 kDa; equine myoglobin, 17 kDa; and vitamin B12, 1.35 kDa) under the same conditions as the MacTopoIIIα.

### Materials

*Methanosarcina acetivorans* C2A genomic DNA was used for amplification of the topoisomerase. *E. coli* JM109 competent cells were used as host cells for amplification of the plasmids pGEM-T and pUC18. The negatively supercoiled pGEM-T and pUC18 DNA were isolated with the Qiagen Mini Plasmid kit according to the specification of the manufacturer and used in the topoisomerase assay as substrate DNA. Reagents for SDS–PAGE, 1 protein molecular weight standards, 1 DNA molecular mass standards, etoposide, camptothecin, EDTA, nalidixic acid, novobiocin, spermidine, ethidium bromide and DTT were purchased from Sigma. HiTrap Q HP, HiTrap S HP, and Superdex 200 HR 10/30 columns were from GE Healthcare. BSA, single-stranded M13 ssDNA was from New England BioLabs. Gel Filtration standards were obtained from Bio-Rad.

## Supporting Information

Figure S1 **Relaxation of negatively supercoiled pUC18 DNA by MacTopoIIIα N1-586 and C587**–**752 at higher concentrations.** Standard reaction mixtures with 90 mM NaCl were incubated with 0.3 µg of pUC18 for 30 min at 37°C with 10 mM MgCl_2_. The only exception is Lane 1 which contains no divalent cation. Lanes 1 and 2 contain 5 pmol of MacTopoIIIα wild-type only. Lane 3 contains 50 pmol of N1-586 only. Lane 4 contains 50 pmol of C587–752 only. Lanes 5–11 contain varying ratios of MacTopoIIIα C587–752 to MacTopoIIIα N1-586 in pmol ranging from 50∶1, to 50∶5 to 50∶10 to 50∶50 to 10∶50 to 5∶50 to 1∶50, respectively. Lanes 12–18 contain varying ratios of MacTopoIIIα C587–752 to MacTopoIIIα N1-586 in pmol ranging from 10∶0.5, to 10∶1 to 10∶5 to 10∶10 to 5∶10 to 1∶10 to 0.5∶10, respectively.
(TIF)
