# Supplementary figures and images for: Methanosarcina acetivorans C2A Topoisomerase IIIα, an Archaeal Enzyme with Promiscuity in Divalent Cation Dependence
Source: PLoS One. 2011 Oct 26;6(10):e26903. doi: 10.1371/journal.pone.0026903 (PMC3202574; doi:10.1371/journal.pone.0026903)

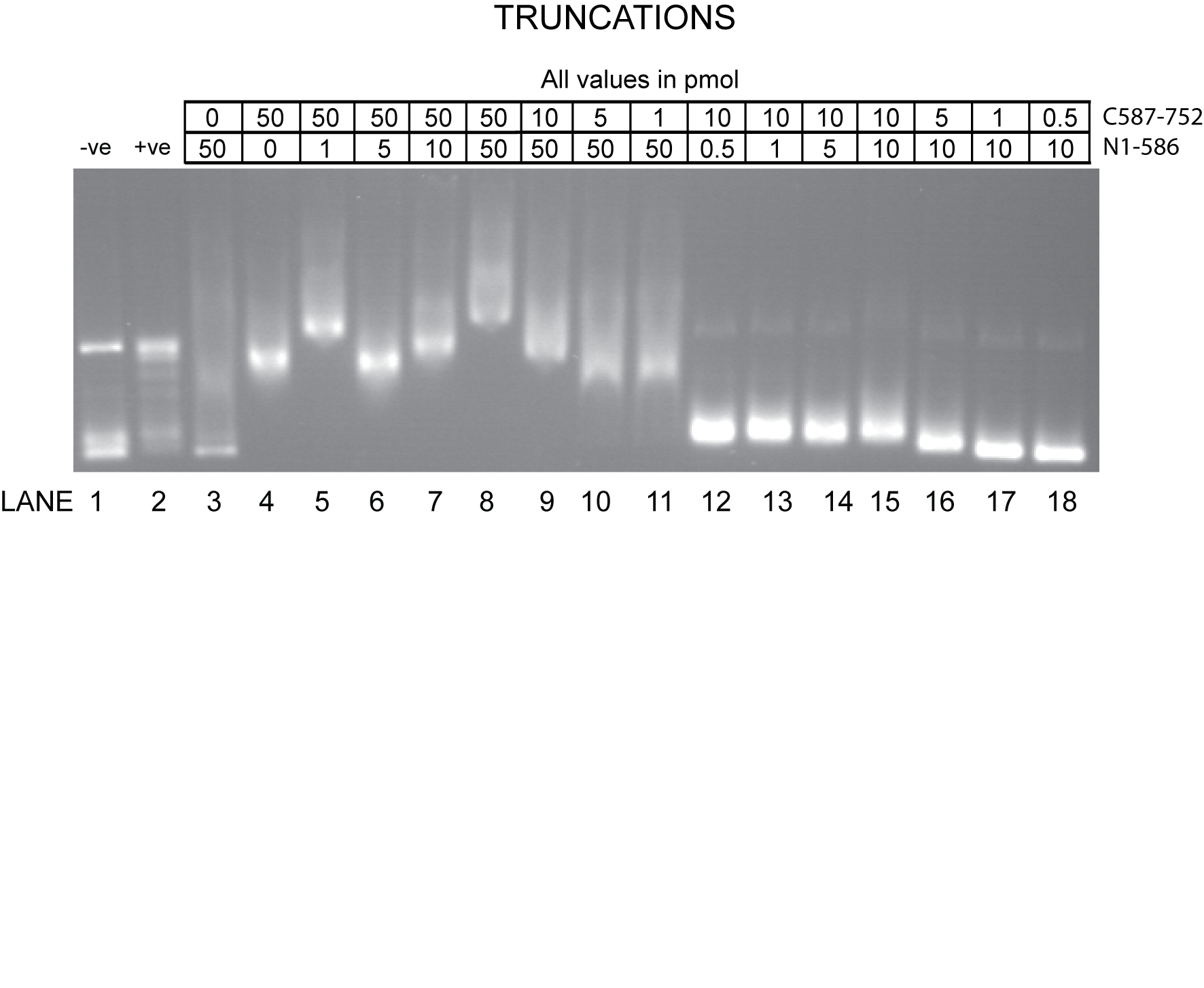

Supplement: Figure S1 — Relaxation of negatively supercoiled pUC18 DNA by MacTopoIIIα N1-586 and C587–752 at higher concentrations. Standard reaction mixtures with 90 mM NaCl were incubated with 0.3 µg of pUC18 for 30 min at 37°C with 10 mM MgCl2. The only exception is Lane 1 which contains no divalent cation. Lanes 1 and 2 contain 5 pmol of MacTopoIIIα wild-type only. Lane 3 contains 50 pmol of N1-586 only. Lane 4 contains 50 pmol of C587–752 only. Lanes 5–11 contain varying ratios of MacTopoIIIα C587–752 to MacTopoIIIα N1-586 in pmol ranging from 50∶1, to 50∶5 to 50∶10 to 50∶50 to 10∶50 to 5∶50 to 1∶50, respectively. Lanes 12–18 contain varying ratios of MacTopoIIIα C587–752 to MacTopoIIIα N1-586 in pmol ranging from 10∶0.5, to 10∶1 to 10∶5 to 10∶10 to 5∶10 to 1∶10 to 0.5∶10, respectively. (TIF) [file pone.0026903.s001.tif]
